# Beneficial Effects of Cocoa Flavanols on Microvascular Responses in Young Men May Be Dependent on Ethnicity and Lifestyle

**DOI:** 10.3390/nu16172911

**Published:** 2024-08-31

**Authors:** Hassan M. Latif, Sophie R. Richardson, Janice M. Marshall

**Affiliations:** School of Biomedical Sciences, Institute of Clinical Sciences, University of Birmingham, Birmingham B15 2TT, UK

**Keywords:** cocoa, flavanols, endothelium, vasodilatation, nitric oxide, hyperaemia, mental stress, acetylcholine

## Abstract

Cocoa flavan-3-ols affect endothelium-dependent responses in resistance vessels and microcirculation has received little attention. We tested the effects of dark chocolate consumption (396 mg total flavanols/day for 3 days) in two Groups of 10 men (18–25 years; non-smokers) each comprising equal numbers of White European (WE) and South Asian (SA) ethnicity. In Group 1, dark chocolate did not affect reactive hyperaemia in forearm muscle, but augmented muscle dilatation evoked by acute mental stress, and reactive hyperaemia and acetylcholine (ACh)-evoked dilatation in cutaneous microcirculation. Conversely, in Group 2, chocolate did not affect cutaneous reactive hyperaemia or ACh-evoked dilatation, but these responses were blunted in Group 1 relative to Group 2. Further, when Groups 1 and 2 were combined, responses were blunted in SAs relative to WEs, augmented by chocolate in SAs only. In Group 2 individuals whose ACh-evoked dilatation was attenuated by nitric oxide synthase (NOS) inhibition, ACh-evoked dilatation was not altered after chocolate, but the attenuating effect of NOS inhibition was lost. Conversely, in Group 2 individuals whose ACh-evoked dilatation was *enhanced* by NOS inhibition, ACh-evoked dilatation was also augmented by chocolate. We propose that in resistance and microvessels of young men, cocoa flavan-3-ols preferentially augment endothelium-dependent dilator responses whose responses are depressed by familial and lifestyle factors more prevalent in SAs than Wes. Flavan-3-ols may facilitate the NOS pathway but also influence other endothelium-dependent dilators.

## 1. Introduction

It is well documented that flavanols, specifically flavan-3-ols, a subgroup of polyphenols abundant in red wine, tea and cocoa, have anti-hypertensive effects and that consumption of cocoa flavanols in particular can reduce resting arterial blood pressure (ABP), especially in hypertensives, acutely and over the longer term [1,2,3,4]. There is also substantial evidence that cocoa flavan-3-ols augment endothelium-dependent dilatation as characterised by Flow Mediated Dilatation (FMD)—the increase in brachial artery diameter caused by the increase in shear stress that follows the release of arterial occlusion [2,3,4]. FMD is largely NO-dependent and is attenuated by NOS inhibition [5]. Accordingly, following acute consumption of high flavan-3-ol-containing cocoa by healthy young men, plasma flavan-3-ols and NO metabolites increased with a similar time course to the augmentation of FMD, reaching a peak at ~2 h and waning by 6 h, while an infusion of NOS inhibitor attenuated the augmentation of FMD [6]. Further, in young male smokers with depressed FMD, not only did acute consumption of high flavan-3-ol-containing cocoa augment FMD, but daily cocoa consumption over 7 days was associated with a progressive increase in FMD such that augmented FMD was present at least 12 h after the last cocoa drink. Plasma nitrite also increased over the 7 days, the daily doses of cocoa inducing further acute increases [3]. These results suggested cocoa flavan-3-ols have both acute and chronic effects on endothelial dilator function in the brachial artery, a conduit artery, which may be mediated by influences on NO synthesis and/or availability [3].

By contrast, little is known of the effects of cocoa flavan-3-ols on endothelium-dependent dilatation in arterial resistance vessels and tissue microcirculation which are important in determining regional vascular resistance, arterial blood pressure and tissue blood flow. In the few published studies, it was shown by using peripheral arterial tonometry, which assesses changes in pulsatile arterial volume of the finger [7], that baseline pulse wave amplitude in men and women (aged 18–72, mean 44 years), some of whom were smokers, was increased by daily consumption of high flavan-3-ol cocoa over 4 days and further increased by the final cocoa dose on day 5 [8]. However, the increase in pulse wave amplitude during reactive hyperaemia on day 5 was similar to day 1 [8]. Infusion of NOS inhibitor reversed the increase in baseline pulse amplitude at day 5 but not the increase during reactive hyperaemia on day 1 or 5, suggesting that cocoa flavan-3-ols have a chronic facilitatory effect on vasodilator tone via the NO pathway, but no acute effect on reactive hyperaemia [8]. By contrast, in a study on healthy young and older men, in which reactive hyperaemia was assessed in forearm muscle by venous occlusion plethysmography and in forearm skin by laser Doppler imaging, reactive hyperaemia was augmented in both circulations by an acute dose of high flavan-3-ol cocoa on day 1, responses being smaller in the older men [4]. However, there was no change in baseline perfusion in either circulation over 14 days of cocoa, and reactive hyperaemia was augmented to a similar extent on days 1 and 14 even when assessed at least 12 h after the last dose [4]. Given reactive hyperaemia is largely endothelium-dependent in both circulations [9,10], these findings indicated that high flavan-3-ol cocoa exerts acute and chronic facilitatory effects on stimulus-evoked endothelium-dependent dilatation in muscle resistance vessels and cutaneous microcirculation but gave no indication of the mechanism(s). Finally, recordings made by venous occlusion plethysmography in young men showed that both resting forearm blood flow and the forearm vasodilator response to acute mental stress were augmented 2 h after consuming high flavan-3-ol cocoa [11]. Given resting vascular tone and muscle vasodilator responses to mental stress are largely NO-dependent [12,13], these findings suggested cocoa flavan-3-ol acutely facilitates tonic and stimulus-evoked NO-dependent dilatation of resistance vessels [11].

In view of this paucity of data on peripheral circulation and the disparities between findings made in groups of different ages, sexes and lifestyles, the primary aim of this study was to test the effects of 3 days of high flavan-3-ol chocolate on changes in forearm vascular resistance and cutaneous microcirculation evoked during reactive hyperaemia by mental stress and endothelium-dependent dilator acetylcholine (ACh) in the same group of young men. In a different group of young men, we tested whether the effects of 3 days of high flavan-3-ol chocolate on ACh-induced dilatation might be explained by effects on the NO pathway. Since ethnicity has not been taken into account so far, and since we recently showed that endothelium-dependent dilatator responses to the chosen stimuli are depressed in those of South Asian (SA) ethnicity relative to White Europeans (WE) [9,14,15], we recruited equal numbers of the two ethnicities into each experimental group: Groups 1 and 2.

## 2. Materials and Methods

This study was approved by the University of Birmingham Ethics Committee (ERN_15-0714). It was not registered in Clinical Trials or any other repository. Young male participants who were apparently healthy were recruited from the student population, or by word of mouth to include only those of SA or WE ethnicity. Ethnicity was self-declared according to criteria used by the Office of National Statistics [16], both parents being of the same ethnicity. Exclusion criteria were hypertension (systolic pressure > 135 mmHg; diastolic pressure > 85 mmHg), tobacco smoking and regular training for high-level competitive sport. Inclusion criteria were that they were normotensive, had no chronic illness including cardiovascular or respiratory disease and were non-smokers. Each completed a lifestyle questionnaire which included information on diet quantified as portions/day of fruit, vegetables, oily fish (as estimates of the antioxidant, cardioprotective vitamins A, C, D, E and omega-3 fatty acids), alcohol (units/week) and caffeinated drinks (cups/day), physical activity, ethnicity and whether or not their parents were hypertensive, had other cardiovascular diseases or diabetes. In Group 1, physical activity was assessed semi-quantitatively, with participants answering questions on time spent/week in moderate exercise (e.g., walking, jogging) and vigorous exercise (running, swimming, rowing, dancing, etc.). The total time was calculated and time spent in vigorous exercise was multiplied by 2. Group 2 completed the International Physical Activity Questionnaire (IPAQ), which allows activity to be quantified as MET (metabolic equivalent of task) min/week where 1 MET represents resting energy expenditure, and walking, moderate and vigorous-intensity exercise are calculated as 3.3, 4.0 and 8.0 MET, respectively, multiplied by their duration per week [17]. The questionnaires were completed at the familiarisation visit to the laboratory when they were shown the recording equipment. Brachial artery blood pressure (ABP) was also recorded at rest with an automated sphygmomanometer (M4, Omron Healthcare, Kyoto, Japan) until three consistent recordings were obtained. At this stage, each participant signed an informed consent form after they had read the participant’s information sheet which gave a full description of the study and had had the opportunity to discuss any queries. Each participant was instructed to refrain from alcohol, caffeine and non-steroidal anti-inflammatory drugs for 12 h preceding each experimental session. They were advised to continue their normal diet over the 3 days as in other studies [2,3,6,8], but to consume only a light breakfast avoiding tea and coffee and flavan-3-ol-containing foods on the day of the laboratory experiment. They were also instructed not to undergo vigorous exercise for 24 h prior to the sessions.

The majority of experiments were carried out on 10 men (21.3 ± 1.8 years; mean ± standard deviation (SD), range 19–22 years; Group 1). Experiments involving NOS inhibition were performed on a second group of 10 men (20.9 ± 3.3 years; mean ± standard deviation (SD), range 19–22 years; Group 2). There were equal numbers of individuals of SA and WE ethnicity in Groups 1 and 2; no individual was included in both Groups 1 and 2.

### 2.1. Experimental Procedures

Experiments were carried out in the laboratory at a room temperature of 21–23 °C. The participants rested in a semi-reclined position 45° from supine on a couch. Pulsatile ABP was continuously monitored from a cuff placed on a finger via a Finapres Nova BP Monitor (Omeda 2300 Finapres^TM^ BP Monitor, Enschede, The Netherlands) and recorded digitally on a computer via Power Lab using LabChart 8 (AD Instruments, Dunedin, Otago, New Zealand). From this recording, mean ABP was computed, together with heart rate (HR). An ECG was recorded from leads attached to each shoulder and the right ankle which was connected to an ECG recorder (Dual Bio Amp: AD Instruments), and thence to LabChart via the PowerLab. Cutaneous perfusion was recorded by Laser Doppler Fluximetry (LDF) via a Perfusion and Temperature monitor (Moor instruments; moorVMS-LDF2, Axminster, UK), which displays perfusion as cutaneous red cell flux (cRCF) in perfusion units (PU) and skin temperature (degrees). The LDF probe was attached to the anterior surface of the forearm by using double-sided adhesive discs or was inserted into a Perspex chamber which it was attached to by adhesive discs and contained acetylcholine (ACh) dissolved in distilled water (see Protocols below). A site was chosen at which baseline cRCF was 10–25 PU with the aim of avoiding underlying larger arterial and venous vessels (see [9]).

Forearm blood flow (FBF) was measured by venous occlusion plethysmography from the non-dominant arm; this technique predominantly measures blood flow in skeletal muscle [18]. A silastic strain gauge containing gadolinium was looped around the widest part of the forearm, secured in place and connected to a plethysmograph unit (EC6, Hokanson Inc., Washington, WA, USA). The forearm was supported at heart level using foam pads under the elbow and near the wrist. An inflation cuff attached to a Rapid Cuff Inflator (Hokanson E20) was wrapped around the upper arm and a paediatric cuff was wrapped around the wrist. For each measurement of FBF, the wrist cuff was inflated to 200 mmHg to exclude blood flow to the hand and prevent hand circulation from interfering with the recording of FBF. The upper arm cuff was rapidly inflated to 50 mmHg and FBF was calculated from the slope of the increase in forearm volume over the first 1–2 heart beats [19]. The cuffs were then deflated.

### 2.2. Experimental Protocols

**Group 1.** Each subject attended the laboratory on two different days separated by at least 4 days for the same protocol. Prior to the second session, a dark chocolate bar (100% Dark Ecuador 50 g, Hotel Chocolat, Royston, UK) was consumed each day for three consecutive days. This chocolate was chosen as it contained the highest flavan-3-ol content of currently available commercial chocolate (792 ± 27 mg/100 g total flavanol, comprising 253 ± 29.4 mg/ 100 g (−)-epicatechin, 205.7 ± 33.6 mg/100 g (+)-catechin, 334 ± 5.9 mg/100 g procyanidins) as tested by Langer et al. [20]. The identity of this chocolate was revealed by personal communication between J.M.M. and corresponding author of ref. [20]. The subjects consumed each chocolate bar at approximately the same time of day, allowing a 24-h period between consumption, with the final bar consumed at least 12 h before the start of the experimental session. Typically, one bar was eaten at 7 p.m. on Monday, Tuesday and Wednesday in preparation for a 9 a.m. session on Thursday. For each session, ABP was continuously recorded by Finapres throughout; other equipment was attached as required by the protocol.

*Reactive hyperaemia in cutaneous circulation.* The LDF probe was attached to the forearm and the rapid inflator cuff wrapped around the upper arm as described above. Baseline recordings of cRCF and ABP were made for 2 min. The upper arm cuff was inflated to 200 mmHg for 2 min and then deflated; cRCF was recorded until it returned to baseline.

*Cutaneous responses to iontophoresis of ACh.* The iontophoresis well was attached to the forearm, filled with ACh (1% in sterile water) and the LDF probe placed in the well to record cRCF. The iontophoresis controller was programmed to deliver six pulses at 100 μA for 20 s each, followed by a final seventh pulse at 200 μA, successive pulses being separated by 60 s intervals (see [9]). Recordings continued for ~20 s after the final pulse.

*Reactive hyperaemia in whole forearm*. The non-dominant arm was arranged for venous occlusion plethysmography. Three baseline recordings of FBF were recorded as described above. The upper arm cuff was then inflated to 200 mmHg for 2 min to occlude arterial inflow into the arm. At 10 s before upper cuff deflation, the wrist cuff was inflated to 200 mmHg to exclude the hand circulation. The upper arm cuff was deflated from 200 mmHg and immediately reinflated to 50 mmHg to allow FBF to be recorded 2–3 s after release of occlusion and then at 15, 30 45, 60 s, 1.5 and 2 min. The wrist cuff was then deflated and a minimum rest period of at least 2 min was allowed.

*Mental Stress: Stroop word-colour conflict test.* A customised 3-min movie was created, designed to initiate and maintain mental stress for its duration. The movie followed a standard design [21], comprising a black background with eight different words describing colours (red, blue, green, yellow, purple, grey, orange and white), each word appearing on the screen for 1.1 s including a 0.1 s fade in and a 0.1 s fade out transitions between words. Each word was coloured in the colour it described or in one of the other colours, for example, blue was shown in red colour. In total, there were 64 combinations of colour and word. The subject was urged to audibly state the colour of the word. During this test, the wrist cuff was occluded at 200 mmHg throughout; FBF recordings were made as described above at 10, 30, 60, 90, 120, 150 and 180 s from the start.

**Group 2.** The subjects came to the laboratory on two occasions and consumed high flavan-3-ol chocolate for 3 days before the 2nd visit as described for Group 1. The following protocol was performed on each occasion.

*Reactive hyperaemia in cutaneous circulation.* With an LDF probe attached to the non-dominant forearm and the rapid cuff inflator wrapped around the upper arm, cRCF was recorded before and following a 2-min period of arterial occlusion as described above.

*Effect of NOS inhibition on ACh-evoked responses in cutaneous circulation.* The LDF probe was then inserted into a well containing 1% ACh as described for Group 1. A baseline recording of cRCF was made for 2 min and then responses were recorded to six pulses of ACh at 100 μA for 20 s each, followed by a seventh pulse at 200 μA, as described for Group 1. Meanwhile, a second iontophoresis well was attached to a different site on the same forearm and filled with the NOS inhibitor nitro-L-arginine methyl ester (L-NAME: 0.27% solution in distilled water) as described previously [15]. The solution was allowed to diffuse into the skin for 30 min and then aspirated from the well. The solution was replaced with ACh (1% solution), an LDF probe was inserted into the well and ACh iontophoresis was repeated. This dose of L-NAME attenuates dilatation induced in the forearm by iontophoresis of ACh, or corticotrophin-releasing factor, which releases NO from mast cells; application of the stereoisomer D-NAME had no such effect [15,22,23].

### 2.3. Data Analysis

All data is expressed as mean ± SD. Responses evoked in cutaneous circulation are expressed as change from baseline (Δ cRCF) in PU by subtracting the baseline value preceding the stimulus from the value extracted at the times indicated above. For responses evoked by ACh, values were extracted over the last 10 s of each 60 s interval between successive pulses and when the response to the final pulse had reached its maximum value. Recordings of FBF made by venous occlusion plethysmography are expressed in absolute values at baseline and specified times during and after the stimuli in mL·100 mL tissue·min^−1^. Forearm vascular conductance (FVC) was calculated as FBF divided by the appropriate mABP when FBF was recorded and expressed in conductance units (CU).

Statistical analyses were performed using GraphPad Prism 10 for macOS. Two-way repeated measures analysis of variance (RM ANOVA) was used to assess differences in reactive hyperaemia and responses evoked by ACh in cutaneous circulation before and after dark chocolate, to test the effects of dark chocolate on changes in FBF evoked during reactive hyperaemia and on ABP, HR, FBF and FVC during mental stress. Post-hoc comparisons were made using Tukey’s or Sidak’s tests as recommended by Prism. In all cases, *p* < 0.05 was considered statistically significant. Power calculations based on previous and pilot data indicated that a group size of 8–10 was sufficient to detect a 20% change in peak reactive hyperaemia in the whole forearm or forearm cutaneous circulations. Furthermore, a 20% change in mean responses evoked by ACh iontophoretic pulses in cutaneous circulation with 80% power at *p* < 0.05 in young men equally matched for SA and WE ethnicities and between SA and WE ethnic groups [9]. Power calculations for responses evoked by ACh using data collected in young men separated into subgroups by whether their parents were hypertensive and showed depressed responses relative to those with normotensive parents, indicated that a subgroup size of 5–6 would be sufficient to detect a change in the mean ACh response of 30% from the control response with 80% power [9].

## 3. Results

### 3.1. Group 1

Anthropometric data for Group 1 are summarised in Table 1. Their age range was between 19–26 years (21.3 ± 0.6 years). Normal body mass index (BMI) range is 18.5–24.9 and 18–23 kg/m^2^ for WE and SA men, respectively [24]: one SA participant had a BMI of 34.72 kg/m^2^ which is classified as obese; other SAs and WEs were within guidelines. One SA and one WE participant had at least one hypertensive parent; three SAs had parents with diabetes and/or coronary artery disease. The score for cardioprotective foods (fruit, vegetables and oily fish) varied between participants, with two (1 SA/1 WE) reporting <1 portion/day. The exercise score varied considerably between individuals, with four subjects (2 SA/2 WE) reporting <4 h/week: their physical activity comprised periods of walking (≤10min/day required for everyday living). The SA participant with high BMI had scores for cardioprotective food and exercise above the mean for Group 1, while his baseline cardiovascular data and evoked responses to the test stimuli were within 1 SD of the mean data for Group 1. Thus, his data were included in the group analyses.

The baselines of cardiovascular variables before and after dark chocolate are shown in Table 2. There was no significant effect of dark chocolate on any variable. It should be noted that the area of skin from which cRCF was recorded after dark chocolate was chosen to have a similar value to that recorded in the control session.

#### 3.1.1. Cutaneous Circulation

**Figure 1:**
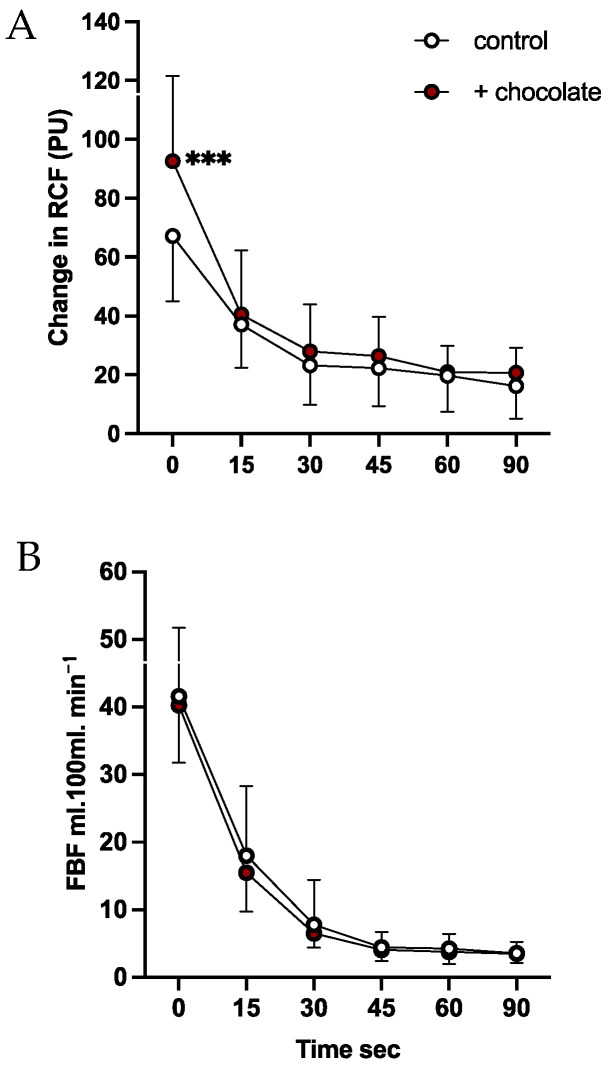
Comparison between effects of dark chocolate on reactive hyperaemia in forearm cutaneous circulation (**A**) and forearm muscle (**B**). Values are shown as mean ± standard deviation (SD). Open and brown-filled symbols are before and after 3 days of dark chocolate, respectively. RCF: cutaneous red cell flux; FBF: forearm blood flow. *** *p* < 0.001: difference between peak values post-hoc Tukey.

*Reactive hyperaemia*. The Δ cRCF recorded during reactive hyperaemia is shown in Figure 1A; the response lasted ~45 s. The Δ cRCF values recorded over the full 90 s showed a main effect of time (*p* < 0.0001), no effect of chocolate (*p* = 0.237), but interaction between time and dark chocolate (*p* = 0.016): post-hoc Tukey showed that peak reactive hyperaemia at time 0 was higher after chocolate than before (*p* < 0.001; Figure 1A).

**Figure 2:**
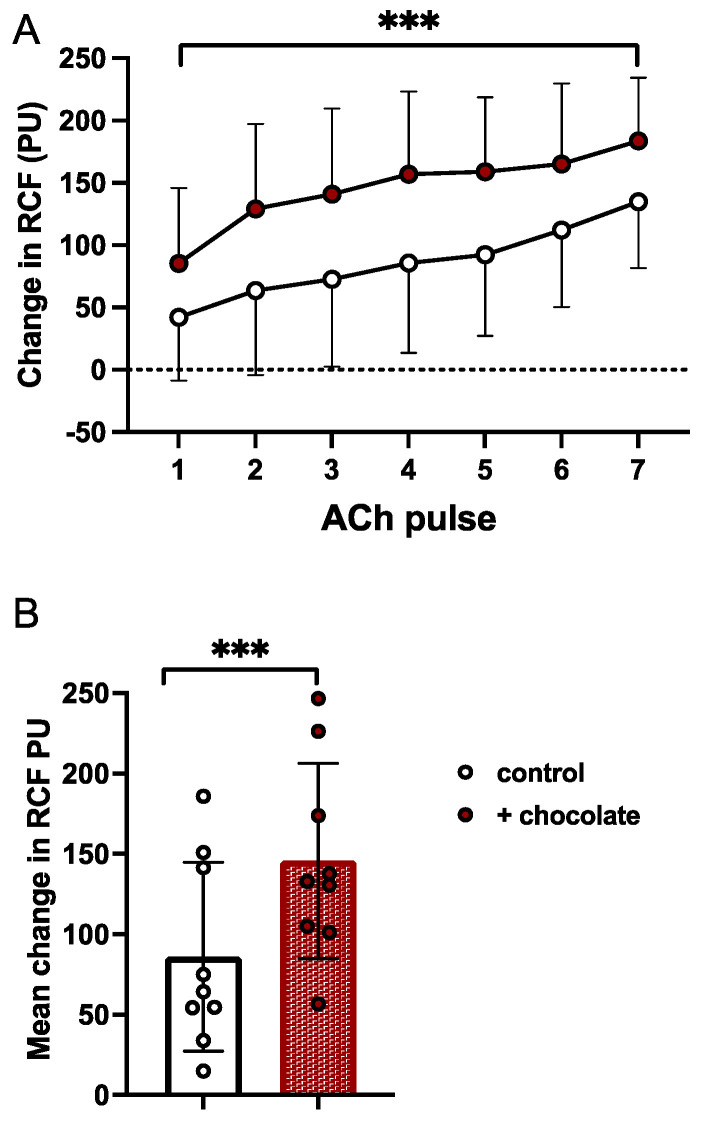
Effects of dark chocolate on responses evoked in forearm cutaneous microcirculation by acetylcholine (ACh). (**A**): changes in cRCF evoked by seven successive iontophoretic pulses of ACh before (open symbols) and after (brown-filled symbols) dark chocolate. Values are shown as mean ± SD. (**B**): open and brown-filled columns show compacted mean values ± standard deviation (SD) for changes in RCF (cutaneous red cell flux) evoked by all seven pulses of ACh before and after dark chocolate. Open and brown-filled symbols represent the mean change in RCF evoked by all seven pulses in individual participants before and after dark chocolate. ***: *p* < 0.001 before vs. after dark chocolate.

*Responses evoked by ACh.* As shown in Figure 2A, ACh evoked a progressive increase in Δ RCF. There was no change in ABP during iontophoresis, so an increase in Δ cRCF indicates cutaneous vasodilatation. The ACh-evoked responses were augmented after dark chocolate: main effects of time, chocolate and interaction were *p* < 0.001, *p* = 0.0472 and *p* = 0.1513, respectively). Figure 2B shows mean responses to all seven pulses of ACh in individual participants before and after dark chocolate, the columns showing the compacted mean values for all individuals under each condition.

#### 3.1.2. Forearm Circulation

*Reactive Hyperaemia.* A substantial increase in FBF was recorded after release of occlusion, with FBF returning to basal levels by 45 s in both conditions (Figure 1B, main effect of time: *p* < 0.001). As there was no change in ABP, this reflected vasodilatation in forearm muscle. Baseline FBF recorded before and after dark chocolate were not significantly different (main effect of chocolate: *p* = 0.122). Although there was no significant effect of chocolate on baseline FBF, we also performed analyses on the Δ FBF from baseline; RM ANOVA showed no effect of chocolate on Δ FBF (*p* = 0.404).

**Figure 3:**
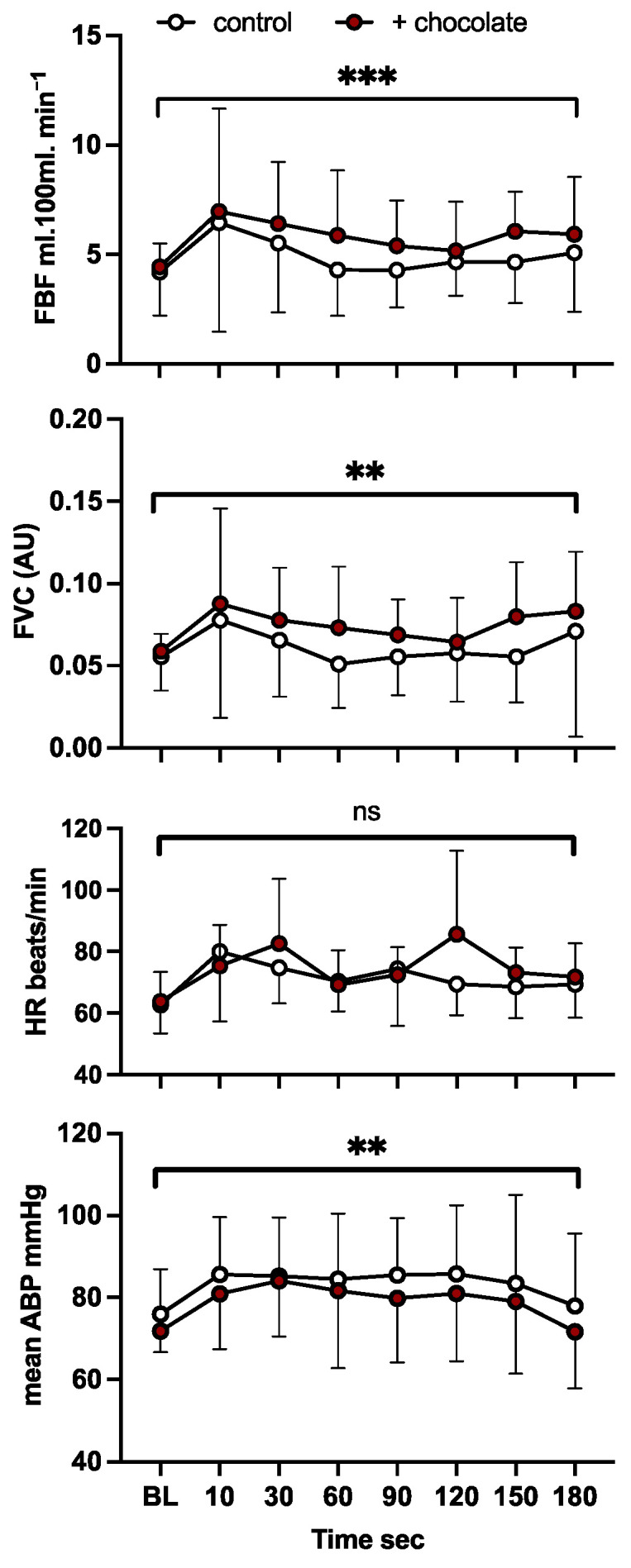
Cardiovascular responses evoked by acute mental stress before and after dark chocolate. From above downwards, forearm blood flow (FBF), forearm vascular conductance (FVC), heart rate (HR) and mean arterial blood pressure (ABP) recorded at intervals during a 3-min Stroop test. Values are shown as mean ± standard deviation (SD) before (open), and after dark chocolate (brown-filled) circles at baseline (BL), 10 s and then 30 s intervals. ***, **: *p* < 0.001, 0.01 respectively before vs. after dark chocolate; ns: *p* > 0.05.

*Forearm and systemic cardiovascular responses evoked by Mental stress.* Before dark chocolate, participants showed an initial increase in mean FBF by 10 s into the Stroop test, accompanied by an increase in mean ABP and HR. Mean FVC also increased by 10 s indicating net vasodilatation in forearm muscle (see Figure 3). Thereafter, FBF and FVC returned towards or below baseline i.e., the forearm vasodilatation was not maintained, and neither was the tachycardia, but mABP was maintained above baseline until the final minute of the Stroop test. By contrast, after dark chocolate, there was a maintained increase in FBF and FVC, with both variables being higher after dark chocolate than before (*p* < 0.001 and *p* = 0.001, respectively). Concomitantly, mABP was lower during Stroop test after dark chocolate than before (*p* = 0.009; Figure 3). There was no effect on HR (*p* = 0.168).

Scrutiny of the FVC data revealed the high variability was explained by some individuals showing an increase in FVC during mental stress, and others showing a fall, indicating forearm vasoconstriction. Thus, further analyses were undertaken on the change in FVC from baseline (Δ FVC) before vs. after dark chocolate. The Δ FVC was different between the control and chocolate conditions (*p* = 0.016), the mean Δ FVC being +0.006 ± 0.009 vs. +0.018 ± 0.009 CU before vs. after chocolate, respectively (Supplementary Materials Figure S1). Three of the five individuals who showed forearm vasoconstriction before chocolate were SAs; most showed less pronounced vasoconstriction or vasodilatation after chocolate. The remainder (2 SAs/2 WEs) showed greater vasodilatation after chocolate, the exception being one SA (Supplementary Materials Figure S1).

### 3.2. Group 2

The anthropometric characteristics of Group 2 were similar to those of Group 1 (Table 3 cf. Table 1). As in Group 1, one SA had a BMI of 25.00 kg·m^2^, higher than the normal range for SA men (see [24]). One SA and one WE had one or both parents with hypertension, but no other cardiovascular disease or diabetes was reported. As in Group 1, the habitual level of physical activity varied between individuals, the data collected from the IPAQ score amongst Group 2 revealed scores ranging from 825.50 to 9906 MET min/week, but only two (1 SA/1 WE) reported low activity levels (<1 h/day walking). Judging from the semi-quantitative analyses of cardioprotective foods, the scores for Group 2 appeared higher than for Group 1 (4.32 ± 2.63 vs. 2.61 ± 1.04 portions/day, respectively), but they were not significantly different by unpaired *t*-test (*p* = 0.068).

As in Group 1, there were no differences between the baseline cardiovascular variables recorded before vs. after dark chocolate (Table 4).

#### 3.2.1. Reactive Hyperaemia in Cutaneous Circulation

In Group 2, following the release of arterial occlusion, Δ cRCF increased to a peak and returned to near baseline by ~45 s. There was a significant main effect of time after occlusion release, but in contrast to Group 1, there was no effect of dark chocolate on Δ cRCF during reactive hyperaemia and no interaction (*p* < 0.001; *p* = 0.576 and *p* = 0.5494, respectively). Peak Δ cRCF before and after consumption of dark chocolate was 71.05 ± 18.51 vs. 78.63 ± 16.44 PU, respectively (see Supplementary Materials Figure S2). In view of the disparity between Groups 1 and 2, we compared reactive hyperaemia *before* dark chocolate between the groups: there was a trend for the response to be greater in Group 2 (*p* = 0.076). When Groups 1 and 2 were combined, there were effects of time, and time x chocolate interaction on reactive hyperaemia, but no effect of chocolate (*p* < 0.0001; *p* = 0.0023 and *p* = 0.1844, respectively). Post-hoc Sidak’s test showed that peak Δ cRCF was higher after chocolate (*p* < 0.0001, see Supplementary Materials Figure S2).

**Figure 4:**
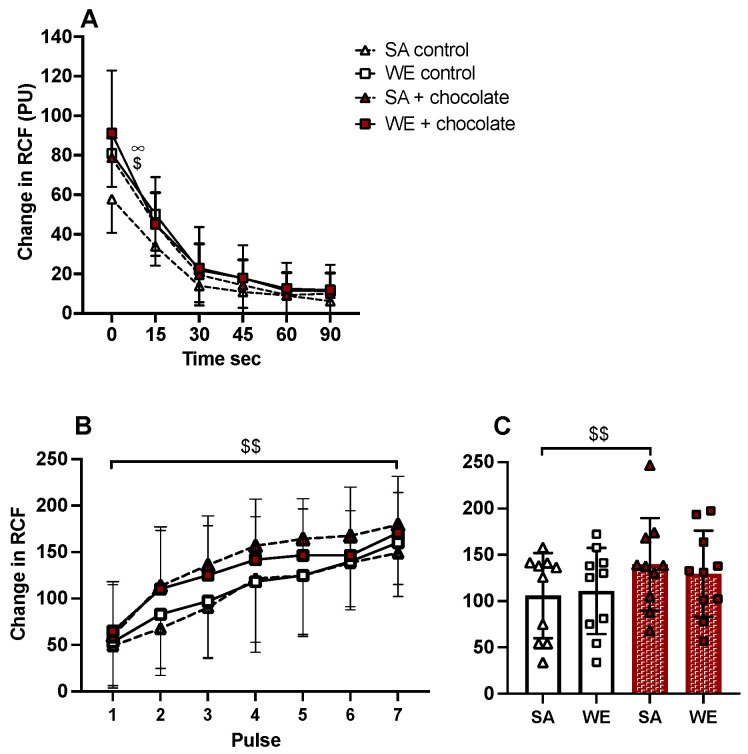
Reactive hyperaemia (**A**) and responses evoked by ACh (**B**) in forearm cutaneous microcirculation of South Asians (SA) and White Europeans (WE) before and after dark chocolate. SAs are shown as triangles and WEs as squares. Values are shown as mean ± standard deviation (SD) before (open) and after dark chocolate (brown-filled) symbols in (**A**,**B**), and as columns (**C**). RCF: cutaneous red cell flux. In (**C**), open and brown-filled symbols represent mean values evoked by all seven pulses of ACh in individual participants before and after dark chocolate. In (**A**), ∞: *p* < 0.05, peak value before chocolate in SAs vs. WEs; $: *p* < 0.05 peak value before vs. after chocolate in SAs. In (**B**,**C**), $$: *p* < 0.01 before vs. after dark chocolate in SAs across all seven pulses of ACh.

To test whether ethnicity might have contributed to variability in reactive hyperaemia in Groups 1 and 2, the effects of dark chocolate and ethnicity were compared across the full groups of participants. RM ANOVA showed interactions between ethnicity and time and before vs. after chocolate, while post-hoc tests showed peak Δ cRCF was greater in WE than SA before chocolate (*p* = 0.0158) and was augmented at peak within SAs after dark chocolate (*p* = 0.045), but not within WEs (*p* = 0.818, see Figure 4A). Peak values for Δ cRCF were 57.75 ± 16.18 and 78.77 ± 9.60 PU before and after chocolate, respectively in SAs, and 80.81 ± 16.02 and 91.19 ± 30.19 PU before and after chocolate, respectively in WEs.

#### 3.2.2. Effect of Chocolate and NOS Inhibition on ACh-Evoked Responses

**Figure 5:**
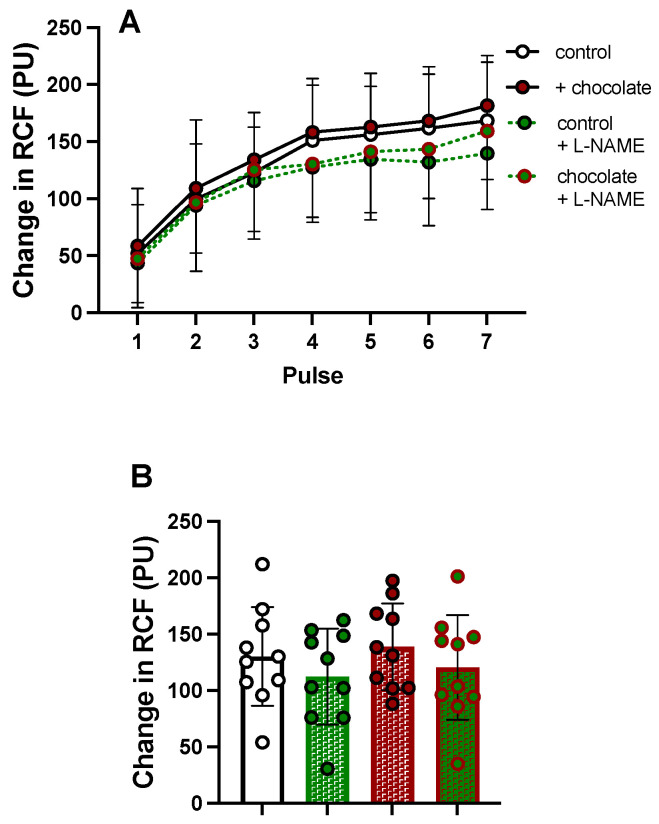
Effect of NOS inhibitor (L-NAME) on responses evoked in forearm cutaneous microcirculation by acetylcholine (ACh) before and after chocolate in Group 2. In (**A**) above, responses evoked by seven pulses of ACh are shown as mean (±SD): control, after dark chocolate, after L-NAME, and after dark chocolate combined with L-NAME as indicated by symbols and continuous/dashed lines. In (**B**), columns show compacted mean values ± standard deviation (SD) for changes in cutaneous red cell flux (RCF) evoked by all seven pulses of ACh under all four conditions, with symbols indicating mean responses in individuals. There was no significant difference between any pair of conditions.

In Group 2, iontophoresis of ACh evoked graded increases in Δ cRCF (Figure 5A). However, in contrast to Group 1, there was no difference between responses evoked before and after dark chocolate (Figure 5A, *p* values for effects of time, chocolate and interaction were *p* < 0.0001, *p* = 0.646 and *p* = 0.971, respectively). Before chocolate, responses evoked by ACh after L-NAME were not significantly different from the control ACh response (*p* = 0.394; Figure 5A). Similarly, after chocolate, the ACh-evoked response after L-NAME was not different from the control response after chocolate (*p* = 0.532; Figure 5A). Figure 5B shows compacted mean values for responses evoked under each condition together with mean changes across all seven ACh pulses in individuals.

In view of the lack of effect of dark chocolate on Group 2, we compared the ACh-evoked response *before* dark chocolate between Groups 1 and 2: it was depressed in Group 1 relative to Group 2 (*p* = 0.0033, Figure 5 control data cf. Figure 2 control data). When Groups 1 and 2 were combined, there were effects of time and chocolate on the ACh-evoked response (*p* < 0.0001 and *p* = 0.0134, respectively), but no interaction (*p* = 0.3049, see Supplementary Materials Figure S3).

To test whether ethnicity might have contributed to the difference between Groups 1 and 2, the effects of dark chocolate and ethnicity were compared across Groups 1 and 2 combined. Control ACh-evoked dilatation was not different between SAs and WEs (*p* = 0.786). However, ACh-evoked dilatation was augmented after dark chocolate in SAs (*p* = 0.002), not WEs (*p* = 0.096; Figure 4B,C).

**Figure 6:**
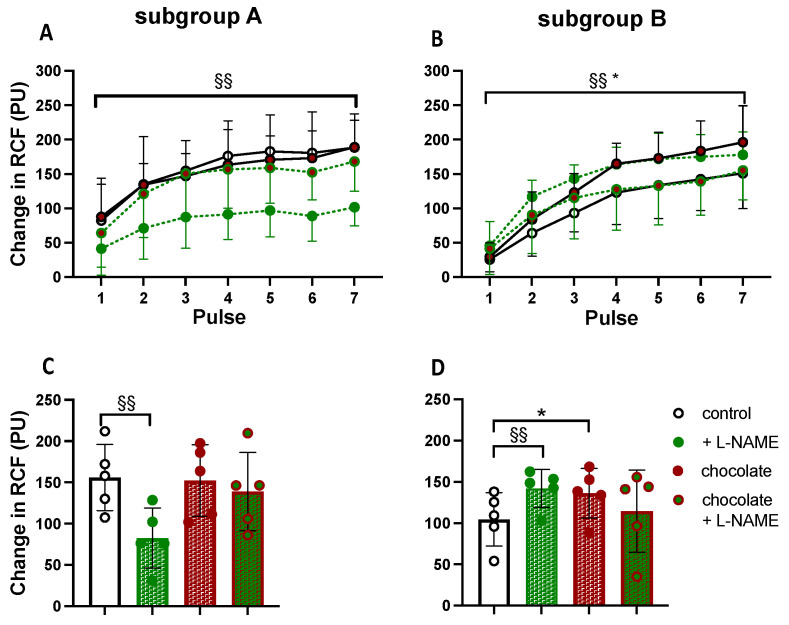
Effect of dark chocolate and NOS inhibitor (L-NAME) on responses evoked in forearm cutaneous microcirculation by acetylcholine (ACh) in subgroup A (Left panel) and subgroup B (Right panel). RCF: cutaneous red cell flux. In (**A**,**B**), responses evoked by seven pulses of ACh are shown as mean (±SD): control, after dark chocolate, after L-NAME, and after dark chocolate combined with L-NAME. In (**C**,**D**), columns show compacted mean values ± standard deviation (SD) for changes in cRCF evoked by all seven pulses of ACh under all four conditions, with symbols indicating mean responses in individuals. Symbols shown in (**D**) apply to (**A**–**D**). *: *p* < 0.05, before vs. after dark chocolate, §§: *p* < 0.01, before vs. after L-NAME.

Considering the data collected in Group 2 *before* dark chocolate, five of the 10 participants showed a reduction in the mean Δ cRCF response to ACh after L-NAME while the remainder showed *augmentation*. Thus, we split Group 2 into subgroups on the basis of the effect of NOS inhibition (subgroup A and B, respectively; see Figure 6) recognising that our power calculations indicated a group size of 5–6 would allow a difference of 30% to be detected from the mean control ACh response with 80% power (see Data analysis).

*Before* dark chocolate, ACh-evoked dilatation was larger in subgroup A than in subgroup B (*p* < 0.001; see Figure 6 right cf. left). In subgroup A (3/5 of whom were SAs), L-NAME attenuated the ACh-evoked response from a mean value of 155.9 ± 36.2 to 82.7 ± 19.1 PU (*p* = 0.015, effect size 47%; Figure 6A,C). However, there was no effect of dark chocolate on the ACh-evoked response in subgroup A (mean response after chocolate was 152.1 ± 31.1 PU; *p* = 0.219) and after chocolate L-NAME did not affect the ACh-evoked responses (mean response was 138.8 ± 33.3 PU; *p* = 0.728 vs. after chocolate, effect size 11%; Figure 6A,C). By contrast, in subgroup B (2/5 of whom were SAs), the ACh-evoked response was *enhanced* by L-NAME *before* chocolate (from 104.6 ± 42.7 to 142.1 ± 44.1 PU; *p* < 0.001, effect size 36%) and the ACh-evoked response was also augmented after dark chocolate (to 136.3 ± 56.4 PU; *p* = 0.002 vs. control response, effect size 30%; Figure 6B,D). After dark chocolate, there was a trend for L-NAME to attenuate the ACh-evoked dilatation (to 114.5 ± 35.2 PU; *p* = 0.056 vs. after chocolate, effect size 16%; Figure 6B,D).

## 4. Discussion

In this study on young healthy men matched for white European (WE) and South Asian (SA) ethnicity, consumption of high flavan-3-ol dark chocolate for 3 days had no effect on baseline ABP, FBF or FVC, or on reactive hyperaemia in forearm muscle. Vascular responses evoked in forearm muscle by mental stress varied between individuals, but they were changed towards greater vasodilation after dark chocolate, while the accompanying increase in ABP was attenuated. Moreover, in cutaneous microcirculation of the forearm, reactive hyperaemia and ACh-evoked dilatation were augmented after dark chocolate but with variability between individuals. Some of this variability was attributable to ethnicity, with SAs showing depressed responses relative to WEs and greater augmentation after dark chocolate. Further analysis indicated that ACh-evoked dilatation was not altered by dark chocolate in those whose control response was NO-dependent but *was* augmented in those whose ACh-evoked dilatation was *not* NO-dependent. After dark chocolate, we detected no effect of NOS inhibition on ACh-evoked dilatation in either subgroup. The factors that may underlie the effects of dark chocolate and variation between individuals are discussed below.

The high flavan-3-ol chocolate used in the study contained 792.8 ± 27.0 mg total flavan-3-ols/100 g (~396 mg/50 g bar [20]): participants consumed one 50 g bar/day for 3 days. Thus, they consumed approximately twice the 200 mg dose of flavan-3-ols recognised by the European Food Safety Authority (EFSA) as a daily dose which helps maintain endothelium-dependent dilatation in the general population as part of a balanced diet [25], but less than the 500 mg/day which produced maximal improvement in FMD and decrease in resting blood pressure in middle-aged normotensive men [2].

### 4.1. Reactive Hyperaemia in the Forearm

Assuming flavan-3-ols improve endothelium-dependent dilatation by facilitating the NO pathway [3,6], our finding that 3 days of high flavan-3-ol chocolate had no effect on peak reactive hyperaemia measured with venous occlusion plethysmography in the whole forearm (i.e. skeletal muscle and skin combined [18]), is not surprising. NOS inhibition had no effect on peak reactive hyperaemia and a small attenuating effect in the recovery period [26], especially in the presence of cyclooxygenase (COX) inhibition which blocks prostaglandin (PG) synthesis [27,28]. Such results suggest NO contributes to the shear stress component of reactive hyperaemia which is triggered by the peak increase in FBF and manifests during recovery, whereas factors arising from ischaemia contribute to the peak itself [10]. The effects of selective antagonists indicate that PGs, adenosine and K^+^ channel opening contribute to the peak and recovery of reactive hyperaemia acting interdependently but with redundancy between them [10,27,28,29]. Thus, discriminating against an augmenting effect of dark chocolate on NO in the recovery phase may not be easy if this is accompanied by a reduction in the influence of other mediators.

Our results contrast with those of Heiss et al. [4] who reported that high flavan-3-ol cocoa augmented peak reactive hyperaemia in the forearm of young men on days 1 and 14 of a chronic dosing regimen. However, Heiss et al. [4] gave no details on when “peak” reactive hyperaemia was measured. Since FBF can only be measured at intervals by using venous occlusion plethysmography, and peak reactive hyperaemia increased from 13.2 ± 0.6 (mean ± SEM) under control conditions to 16.2 ± 1.0 or 16.9 ± 0.6 mL/100 mL·min^−1^ on day 1 and 14, respectively in their study, much smaller than our peak values of 41.63 ± 9.6 and 40.24 ± 2.68 mL/100 mL·min^−1^ before vs. after dark chocolate, it is likely their first measurement was made in the recovery period when shear stress contributes to reactive hyperaemia [10]. As the dose (900 mg total flavan-3-ols/day [4]) was twice the dose we used, this may explain why they detected an effect of flavan-3-ols on the recovery component and we did not.

### 4.2. Forearm Vascular Response in Acute Mental Stress

Net forearm vasodilatation evoked by the Stroop test was augmented after 3 days of high flavan-3-ol chocolate. In individuals, forearm vasodilatation was enhanced in those who already showed vasodilatation and converted towards vasodilatation in those who showed forearm vasoconstriction. This extends the finding that net forearm vasodilatation evoked by a mental stress test was acutely augmented 90 min after cocoa containing 680 mg total flavan-3-ols [11].

The direction of the forearm vascular response to acute mental stress depends on the balance between the vasoconstrictor effect of increased muscle sympathetic nerve activity which occurs in most individuals [12,30] and vasodilatation which is endothelium- and NO-dependent [12,13]. The direction of change varies *between* individuals, but the direction and magnitude of changes in sympathetic activity and vascular responses are consistent *within* individuals on repetition of the stress stimulus both within session and across two sessions [14,31]. The vasodilatation has been attributed to circulating adrenaline acting on beta-adrenoreceptors [21] and increased shear stress evoked by the pressor response or tachycardia which augment NO-dependent dilatation [21,32,33]. It is depressed in those whose endothelium-dependent dilatation is blunted and at higher risk of future CVD, for example, in borderline and established hypertensives, Black African Americans and South Asians, partly due to a decreased contribution of NO [14,34,35,36,37,38].

Against this background, the present results are consistent with the idea that high flavan-3-ol chocolate promotes the forearm vasodilator component of the response to mental stress in young men, including those whose endothelium-dependent dilatation is depressed and consistent with a facilitatory effect of flavan-3-ol on the NO pathway [3,6]. Dark chocolate also attenuated the pressor response to mental stress, consistent with evidence that the pressor response and forearm vasodilatation are inversely and causally related [14,35]. Since enhanced pressor responses to stressors in young individuals are associated with an increased risk of CVD [39], our results add to the evidence that dietary flavan-3-ols reduce the risk of future CVD [25].

### 4.3. Reactive Hyperaemia in Forearm Cutaneous Circulation

In contrast to our findings on reactive hyperaemia in the whole forearm, peak reactive hyperaemia in the forearm cutaneous circulation was augmented after 3 days of dark chocolate in Group 1 but not Group 2. The disparity between Groups was unexpected given they were all healthy individuals, matched for age and WE/SA ethnicity. However, there was a trend for reactive hyperaemia to be depressed in Group 1 relative to Group 2 (*p* = 0.076). Further, when Groups 1 and 2 were combined, reactive hyperaemia was augmented in SAs but not WEs. Endothelium-dependent dilatation is depressed in young SA, relative to WE men [9,14,40]. Thus, the findings indicate cocoa flavan-3-ols may be particularly beneficial in improving endothelium-dependent dilatation in individuals in whom it is depressed. This is discussed further in relation to ACh-evoked cutaneous dilatation (see below).

That peak cutaneous reactive hyperaemia was augmented in some young men after 3 days of dark chocolate compares favourably with the report that in young men of unspecified ethnicity, reactive hyperaemia recorded by laser Doppler imaging was augmented after 14 days of high flavan-3-ol cocoa [4]. We cannot directly compare our results with the report that in the finger, pulse wave amplitude during reactive hyperaemia was augmented after 4 days of high flavan-3-ol cocoa [8]. That study was performed on men and women aged 18–72 years, but recordings of pulse wave amplitude were made 1 min after release of arterial occlusion when cRCF recorded by Laser Doppler Fluximetry in forearm has returned to baseline (Figure 1B, see also [29]). The time course of cutaneous reactive hyperaemia is longer in the finger than in the forearm, probably reflecting the special morphology of finger circulation [41].

Cutaneous reactive hyperaemia in the forearm is partly mediated by PGs judging from the attenuating effect of COX inhibition in some but not all studies on men and/or women [9,15,41,42,43]. It is also partly dependent on NO judging from the attenuating effect of NOS inhibition in young WE and SA women [15], although others found no effect in men and women [42]. However, COX products inhibit the contribution of NO to cutaneous reactive hyperaemia [44]. Thus, the augmenting effect of dark chocolate on reactive hyperaemia in some individuals is consistent with the facilitation of the NO pathway [3,6], but also with flavan-3-ol-induced facilitation of the contribution of PGI_2_ synthesised by COX [45].

### 4.4. ACh-Evoked Cutaneous Vasodilatation

ACh is the classic endothelium-dependent dilator which acts via NO, PGs and endothelium-dependent hyperpolarising factors (EDHFs, [46]). Given that flavan-3-ols facilitate the NOS pathway [3,6], we hypothesised that dark chocolate would augment ACh-evoked dilatation in WE and SA men, and that the contribution of NO would be enhanced after dark chocolate and revealed by a greater effect of NOS inhibition. ACh-evoked dilatation was augmented after dark chocolate in Group 1, but not Group 2, similar to the disparity for reactive hyperaemia. Further, in Group 2, there was no obvious effect of NOS inhibition on ACh-evoked dilatation before, or after dark chocolate. These results not only indicate substantial variation between individuals in the effects of flavan-3-ols on ACh-evoked dilatation but also suggest they are unlikely to be mediated solely by NO. Indeed, similar to our findings for reactive hyperaemia, the control response to ACh was blunted in Group 1 relative to Group 2. Further, when we combined Groups 1 and 2, dark chocolate enhanced ACh-evoked dilatation in SAs, but not WEs. Given that the contributions of NO and PG to ACh-evoked dilatation were impaired in young SA men and women relative to WEs [9,15], these findings are consistent with the proposal made above concerning reactive hyperaemia: that cocoa flavan-3-ols are more effective in augmenting endothelium-dependent dilatation in individuals whose responses are blunted.

The question arises as to why there was so much variation between Groups 1 and 2 since they were recruited from a narrow age-range, matched for ethnicity as well as the criteria usually adopted for studies on healthy individuals (e.g., non-smoking, normotensive, no medication). The additional information we collected on family history and lifestyle may be of significance. Although one SA and WE in each group had hypertensive parents, as many as three SAs in Group 1 had at least one parent with diabetes; none in Group 2. The score for cardioprotective food consumption tended to be lower in Group 1 than 2, and physical activity levels were also lower in Group 1: 4/10 individuals undertaking very little exercise. These points suggest that by chance, the family history and lifestyle of young SA and WE men recruited into Group 1 may have led to relatively depressed endothelium-dependent dilator responses that were more susceptible to the beneficial effects of cocoa flavanols. With the *proviso* that our power calculations indicated subgroup sizes of five would allow us to detect at most, a change in mean ACh response of 30% with a power of 80%, the outcomes when we divided Group 2 according to the effect of NOS inhibition are also consistent with that proposal. Subgroup A whose control ACh-evoked dilatation was *attenuated* by ~47% by NOS inhibition showed greater ACh-evoked dilatation than subgroup B whose ACh-evoked dilatation was *augmented* by ~35% by NOS inhibition and it was only in subgroup B that dark chocolate augmented ACh-evoked dilatation (by ~30%).

Considering the mechanisms that may underlie these differences, the literature reveals considerable variation between studies in the extent to which NO or PGs may contribute to ACh-evoked dilatation in forearm cutaneous circulation. In some studies on men and women, ACh-evoked dilatation was attenuated by NOS inhibition [47,48]; in others, it was not [49,50,51]. We previously found that NOS inhibition had a weaker attenuating effect on ACh-evoked dilatation in young SA than WE women and no net effect in SA women with hypertensive parents. NOS inhibition *augmented* ACh-evoked dilatation in some individuals, particularly those with hypertensive parents [15], a status which confers a higher risk of hypertension and CVD [52]. On the other hand, COX inhibition attenuated ACh-evoked dilatation in some studies on men and women [47,49,50,51], but not in others [43,53]. Moreover, ACh-evoked dilatation was attenuated by COX inhibition in young WE and SA men and women, but again the effects were greater in WEs, weak in those with hypertensive parents, and COX inhibition augmented the dilatation especially in those with hypertensive parents [9,15]. Finally, ACh-evoked dilatation still occurred after combined NOS and COX inhibition, suggesting mediation by EDHF/s [15,51]; both epoxyeicosatrienoic acids (EETs) and opening of calcium-activated potassium (K^+^_Ca_) channels have been implicated [54,55].

Against this backdrop, and acknowledging low statistical power, in subgroup A, the fact that NOS inhibition greatly attenuated ACh-evoked dilatation before dark chocolate agrees with some previous evidence [15,47,48]. If these individuals achieved near maximal dilatation to ACh *before* dark chocolate, as indicated by the plateau of the response (see Figure 6A), then our finding that dark chocolate had no effect on it could be consistent with cocoa flavan-3-ols facilitating the NOS or COX pathways [3,6,45]. Further, as the effect of NOS inhibition on ACh-evoked dilatation was essentially lost *after* dark chocolate, this could be explained if NOS inhibition removed an inhibitory effect of NO on a COX pathway which had been facilitated by cocoa flavan-3-ols [45,56,57], allowing PGs to maintain the ACh-evoked dilatation [9,49,50].

For subgroup B, our finding that NOS inhibition *augmented* ACh-evoked dilatation could be explained if the inhibitory effect of NO on the COX and/or EDHF pathways [56,58] were removed, allowing the contribution of PGI_2_ and/or EDHFs to be revealed [9,49,50,54,55]. The finding that ACh-evoked dilatation was augmented by dark chocolate would be consistent with flavan-3-ols facilitating the NOS and/or COX pathways [3,6,45] or generating EDHFs. Further, the trend for NOS inhibition to have an attenuating effect on ACh-evoked dilatation after dark chocolate (*p* = 0.056) in the context of low statistical power, raises the possibility that in individuals with depressed endothelium-dependent dilatation, dark chocolate augmented ACh-evoked dilatation at least in part by augmenting the NOS pathway and revealing a dilator effect of NO; with NOS inhibited, PGs and/or EDHFs maintained the dilatation [9,49,50].

### 4.5. Limitations of Study

Given the variability in the effects of dark chocolate on ACh-evoked dilatation in cutaneous microcirculation between and within Groups 1 and 2 and between subgroups A and B which comprised only five participants, it is clear that studies should be performed on much larger groups of young men. Such studies should not only test the effects of NOS inhibition before and after dark chocolate but also the effects of COX inhibition alone and combined with NOS inhibition, to assess whether COX products do indeed contribute to the effects of flavan-3-ols in ACh-evoked dilatation as we have proposed. Studies involving the inhibition of EETs and K^+^_Ca_ channels [54,55] could be used to test whether flavan-3-ols affect the contribution of EDHFs.

In view of our proposals that in young men, the variability of endothelium-dependent microcirculatory responses and effects of cocoa flavan-3-ols on those responses is associated with habitual exercise levels, cardioprotective food consumption, familial hypertension, diabetes and SA rather than WE ethnicity, future studies should collect more detailed quantitative information on exercise levels and nutrient consumption including flavan-3-ols than we achieved. Since insulin resistance was identified in young healthy SA men in the absence of diabetes [40] and is an early marker of endothelial dysfunction and risk of CVD in young adults [40,59], plasma insulin and fasting glucose should also be assessed. Together, this would allow a more complete analysis of the profile of young men whose endothelial function would benefit from higher flavan-3-ol levels in the diet. Clearly, similar studies should be performed on young women, recognising that the effects of cocoa flavan-3-ols may vary with the menstrual cycle: the NOS and COX pathways are facilitated by oestrogen [60]. We note that the importance of understanding inter-individual variability in cardiometabolic responses to plant-based dietary nutrients is currently attracting considerable attention (see [61]).

It might be argued that we should have randomised the order of our test stimuli. However, control responses before dark chocolate in this study were of similar magnitude to those recorded in previous studies in which we used different combinations of the stimuli (see [9,14]). Moreover, we purposely tested responses evoked by mental stress at the end of the protocol because acute mental stress impairs endothelium-dependent FMD for ~90 min, recovering by 4 h [62], and high flavan-3-ol cocoa counteracts such effects [11]. Nevertheless, it is a limitation that we did not perform a crossover trial using a low flavan-3-ol chocolate matched for other nutrients, or perform a parallel study with low flavan-3-ol chocolate, so as to differentiate flavan-3-ol and placebo effects.

## 5. Conclusions

On the basis of our results, we suggest that in young SA and WE men, high flavan-3-ol chocolate consumed daily for 3 days can augment endothelium-dependent dilatation in the forearm muscle and attenuate the associated pressor response to acute mental stress, as well as augment reactive hyperaemia and ACh-evoked dilatation in cutaneous microcirculation. The dilator responses were augmented most in those whose endothelium-dependent dilatation was depressed, a condition which seemed attributable to lifestyle factors more prevalent in young SA than WE men. Since muscle vasodilator and pressor responses to acute mental stress are associated with the risk of hypertension and CVD [39], while endothelium-dependent dilator responses in cutaneous microcirculation are associated with the risk of coronary artery disease [63], it seems reasonable to propose that increasing dietary levels of flavan-3-ols should reduce the risk of these disorders in SA and WE men. Mechanistically, our results accord with flavan-3-ols augmenting endothelium-dependent dilatation in resistance vessels and microcirculation in part via the NO pathway, as proposed for FMD [6]. However, aspects of our results indicate that the effects of cocoa flavan-3-ols on the pathways that generate PGs [45] and EDHFs also require attention.

## Figures and Tables

**Table 1:** Anthropometric characteristics of Group 1 (*n* = 10).

	WEs	SAs	All
Age (years)	21.0 ± 0.63	21.6 ± 2.4	21.30 ± 1.8
BMI (kg/m^2^)	24.30 ± 3.05	27.1 ± 4.1	25.70 ± 3.9
Alcohol intake (units/week)	9.8 ± 6.18	1.0 ± 2.0	5.40 ± 2.12
Caffeine (cups/day)	0.50 ± 0.7	1.0 ± 1.26	0.75 ± 0.3
Fruit/vegetable consumption (portions/day)	2.83 ± 1.16	2.44 ± 0.84	2.61 ± 1.04
Oily fish portions/week	2.0 ± 1.0	1.2 ± 0.3	1.7 ± 0.9
Physical Activity level (score/week) *	8.30 ± 5.17	9.0 ± 6.51	8.65 ± 5.88
Parent/s with hypertension (number)	1	1	2
Parent/s with T2D (number)		3	3

All values are given as mean ± standard deviation (SD), except the number of participants with hypertension or Type 2 diabetes (T2D). BMI: Body Mass Index. * Physical activity score is calculated as the number of hours of exercise/week, high intensity exercise being multiplied by 2 (see text for details).

**Table 2:** Baseline cardiovascular variables in Group 1 before and after dark chocolate.

Variable	Control	After Chocolate	*p* Value
SBP (mmHg)	122.0 ± 10.7	120.9 ± 7.1	0.638
DBP (mmHg)	71.5 ± 6.2	69.6 ± 7.2	0.155
MABP (mmHg)	88.3 ± 6.4	86.7 ± 5.5	0.126
HR (beats/min)	66.9 ± 8.49	69.02 ± 10.1	0.380
cRCF (PU)	23.33 ± 9.87	26.01 ± 3.53	0.645
FBF (mL·100 g^−1^·min^−1^)	4.45 ± 1.92	4.33 ± 1.02	0.835
FVC (AU)	0.061 ± 0.021	0.057 ± 0.012	0.622

All values are given as mean ± standard deviation (SD). SBP, DBP and MABP are systolic, diastolic and mean arterial blood pressure, respectively. All other abbreviations are as indicated in text. *p* value: comparison between values recorded before and after dark chocolate by Student’s paired *t*-test.

**Table 3:** Anthropometric characteristics of Group 2 (*n* = 10).

	WEs	SAs	All
Age (years)	21.0 ± 0.6	20.8 ± 0.4	20.9 ± 1.1
BMI (kg/m^2^)	21.6 ± 0.8	23.2 ± 0.6	22.5 ± 1.6
Alcohol intake (units/week)	11.8 ± 2.5	9.7 ± 2.6	10.8 ± 5.8
Caffeine (cups/day)	1.8 ± 1.1	0.4 ± 0.2	1.1 ± 1.9
Fruit/vegetable (portions/ day)	3.8 ± 1.0	4.3 ± 0.8	4.1 ± 2.0
Oily fish portions /week	1.8 ± 1.1	1.6 ± 0.6	1.6 ± 2.1
Physical Activity (MET min/week) *	3042 ± 321	2579. ± 293	2811 ± 2094
Parent/s with hypertension (*n*)	0	1	1
Parent/s with T2D (*n*)	0	0	0

All values are given as mean ± standard deviation (SD), except the number of participants with hypertension or Type 2 diabetes (T2D). BMI: Body Mass Index. * Physical activity scores are calculated from IPAQ (see text) and expressed relative to MET (metabolic equivalent of task/week).

**Table 4:** Baseline cardiovascular variables in Group 2 before and after a 3-day consumption of dark chocolate.

Variable	Control	After Chocolate	*p* Value
SBP (mmHg)	122.1 ± 7.8	119.3 ± 7.5	0.138
DBP (mmHg)	76.5 ± 5.4	74.1 ± 4.5	0.355
MABP (mmHg)	91.7 ± 5.7	89.0 ± 6.3	0.126
HR (beats/min)	77.4 ± 6.5	73.4 ± 6.4	0.680
RCF (PU)	14.33 ± 7.87	11.7 ± 6.25	0.445
FBF (mL·100 g^−1^·min^−1^)	6.75 ± 2.42	6.33 ± 2.42	0.435
FVC (AU)	0.092 ± 0.031	0.084 ± 0.027	0.822

All values are given as mean ± standard deviation (SD). SBP, DBP and MABP are systolic, diastolic and mean arterial blood pressure, respectively. HR: heart rate; RCF: cutaneous red cell flux; FBF: forearm blood flow, FVC: forearm vascular conductance; *p*-value: comparison between values recorded before and after dark chocolate by Student’s paired *t*-test.

## Data Availability

The raw data supporting the conclusions of this article will be made available by the authors on request.

## Supplementary Materials

The following supporting information can be downloaded at https://www.mdpi.com/article/10.3390/nu16172911/s1: Figure S1: Changes in forearm vascular conductance (FVC) during acute mental stress before and after dark chocolate; Figure S2: Effects of dark chocolate on reactive hyperaemia in forearm cutaneous circulation in Group 2 and Groups 1 and 2 combined; Figure S3: Effects of dark chocolate on responses evoked by ACh in Groups 1 and 2 combined.
