# Supplementary material for: Beneficial Effects of Cocoa Flavanols on Microvascular Responses in Young Men May Be Dependent on Ethnicity and Lifestyle
Source: Nutrients. 2024 Aug 31;16(17):2911. doi: 10.3390/nu16172911 (PMC11403714; doi:10.3390/nu16172911)
Supplement: Supplementary file 1 [file nutrients-16-02911-s001.zip › nutrients-3081553-supplementary.pdf]

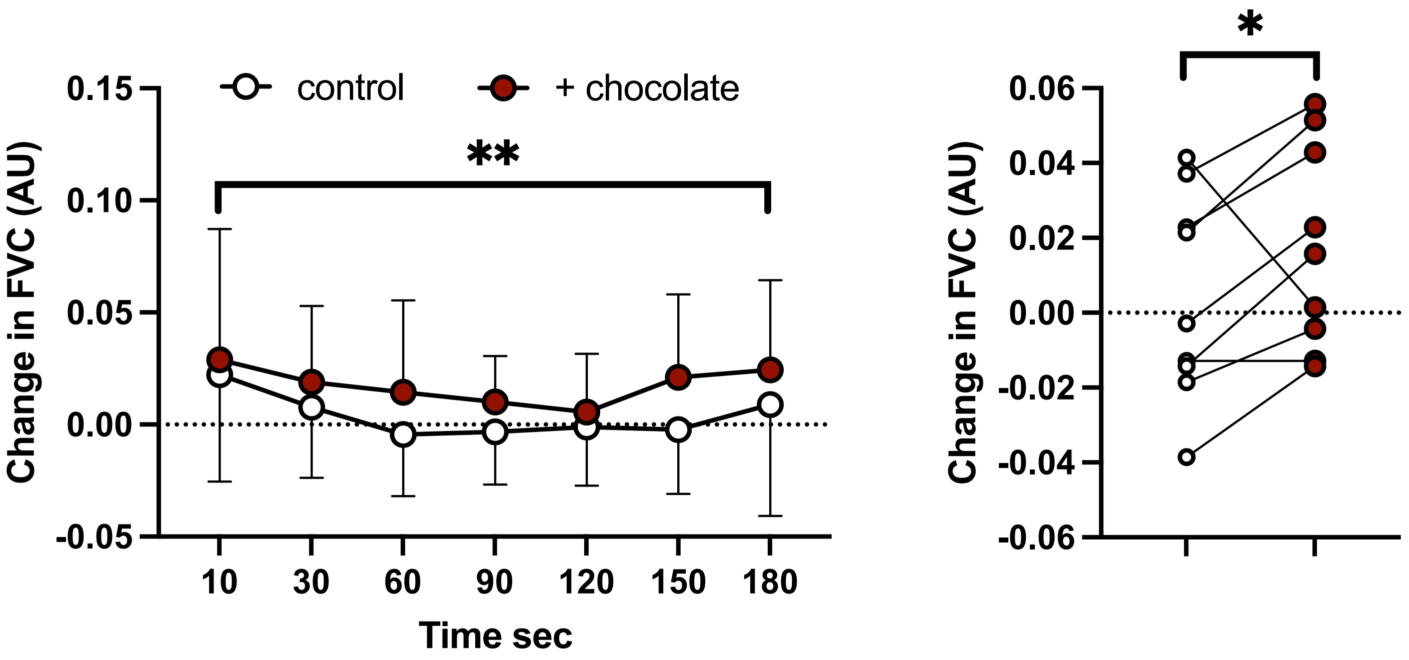

Supplementary Figure S1. Changes in forearm vascular conductance (FVC) during acute mental stress before and after dark chocolate. Open and brown-filled symbols indicate values recorded before and after dark chocolate respectively. Left panel: each value is mean change in FVC from baseline ± SD, at 10s from beginning of Stroop test and then at 30 s intervals for 3 min. Right panel shows mean change in FVC over 3 min Stroop test in individual participants: values recorded before and after dark chocolate are joined by continuous line. **, * P<0.01, 0.05 respectively before vs after dark chocolate. In each panel values shown above dotted line represent forearm vasodilatation, those below line represent vasoconstriction.

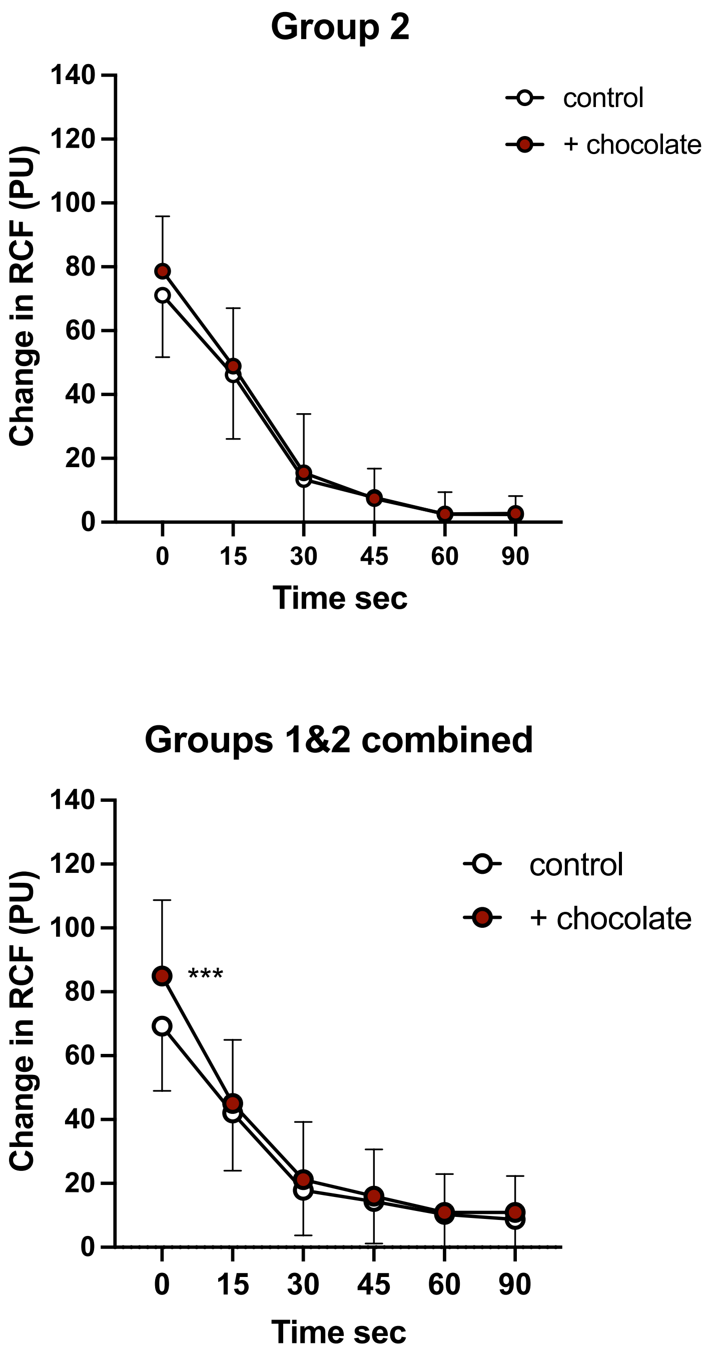

Supplementary Figure S2. Effects of dark chocolate on reactive hyperaemia in forearm cutaneous circulation in Group 2 (above) and Groups 1 and 2 combined (below). Values are shown as mean ± SD. Open and brown-filled symbols: before and after 3-days of dark chocolate respectively. *** P<0.001: difference between peak values by post-hoc Tukey.

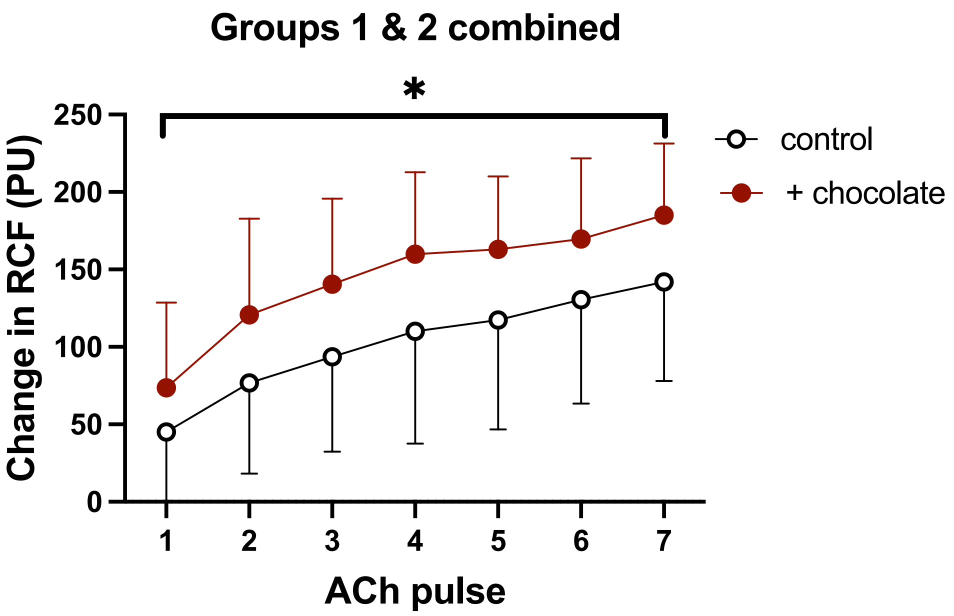

Figure S3. Effects of dark chocolate on responses evoked in forearm cutaneous microcirculation by acetylcholine (ACh) in groups 1 and 2 combined . Changes in cRCF evoked by seven successive iontophoretic pulses of ACh before (open symbols) and after (brown-filled symbols) dark chocolate. Values are shown as mean ± SD. *: $p < 0.05$ before vs. after dark chocolate.
